# Health literacy and behaviors influencing blood sugar level control among type 2 diabetes patients in primary care units, Thailand: A cross-sectional study

**DOI:** 10.12688/f1000research.74225.1

**Published:** 2022-03-18

**Authors:** Atthawit Singsalasang, Rachanon Nguanjairak, Tongtip Salawonglak

**Affiliations:** 1 Faculty of Public Health, Nakhon Ratchasima Rajabhat University, 340 Sura Narai Rd, Tambon Nai Mueang, Nakhon Ratchasima, 30000, Thailand

**Keywords:** Health literacy, blood sugar level control, type 2 diabetes, primary care unit, Thailand

## Abstract

**Background:** Type 2 Diabetes Mellitus (T2DM) remains a significant cause of death globally. In addition, T2DM is among the top five chronic diseases which leads to mortality in the Thai population. Patients with T2DM need a wide self-management protocol.  However, patients with low health literacy experience difficulty in recognizing health-related information and have difficulties in expressing their status to health care providers, resulting in poor self-management which results in worsening of the health condition. This study aimed to identify the health literacy among patients with type 2 diabetes to determine its association between factors with blood sugar level control in the patients who are treated in primary care units.

**Methods:** A total of 605 subjects were randomly selected from four districts of Nakhon Ratchasima Province, Northeastern Thailand. Data were collected using a structured questionnaire and a review of their charts. A descriptive statistical analysis was used to describe characteristics of the subjects. In addition, multiple logistic regression was used for the association to estimate effect sizes in terms of an odds ratio with the 95% confidence interval.

**Results:** Of the total 605 respondents, 90.90% of the subjects had a sufficient level of health literacy about diabetes. The present study found the subjects who had sufficient health literacy were significantly associated with blood sugar level control (Adjusted Odds Ratio, (AOR)=2.27; 95% CI: 1.10-4.74; p =0.026). A strongly significant association with blood sugar level control was found with diet behaviors (AOR = 9.71; 95% CI: 5.98-15.77; p<0.001) and exercise behaviors (AOR = 14.50; 95% CI: 8.66-24.27; p<0.001).

**Conclusions:** Health literacy on the changing health related behaviors among the T2DM patients is significantly associated with controlling blood sugar level. Hence, health practitioners should enhance the health literacy on self-care among T2DM patients which will help to control T2DM in an effective manner.

## Introduction

The complications of Type 2 Diabetes Mellitus (T2DM) are significant causes of death worldwide. ^1^ There was a 5% increase in premature mortality from diabetes in lower-middle-income countries between 2000 and 2016. ^1^ A statistic from the International Diabetes Federation (IDF) reported that the number of people living with diabetes is expected to increase to approximately 642 million people in 2040. ^1^ Similarly in Thailand, T2DM is one of the top five chronic diseases which leads to mortality among populations of the whole country. ^1^^,^^2^ Approximately 17.5% of the patients develop severe complications and at the end-stage they develop chronic kidney diseases. ^1^^,^^2^ A health survey in the Thai population reported the prevalence of diabetes mellitus among people aged 15 years and over, which was increased from 6.9% to 8.96% between 2004 and 2014. In addition, T2DM was found in 9.80% of females and 7.80% of males and the highest prevalence was found among the population aged 60-69 years in 2014. ^3^ It has been estimated that the prevalence of diabetes mellitus will be unraised and constant at 6.90% by the year 2025. ^4^ The mortality statistics also emphasized that the T2DM will be raised in future despite the trend of subsiding the non-communicable diseases. In addition, the highest morbidity and mortality rate are identified with 6 diseases, namely diabetes mellitus, cardiovascular and cerebrovascular diseases, emphysema, cancer, hypertension, and obesity. ^1^ Furthermore, single, frequent complications of diabetes mellitus include chronic kidney disease (CKD) which is found up to 17.50% of diabetes mellitus patients. ^1^ After proceeding to end-stage renal disease (ESRD), patients must take the renal replacement therapy. ^2^

A study of the circumstances of Non Communicable Diseases (NCDs) in Nakhon Ratchasima province in 2016 found that 134,807 cases of diabetes patients represented the public health problem of Nakhon Ratchasima. ^5^ At present, Nakhon Ratchasima health provincial office has arranged primary care units for providing health care service continuously for diabetes mellitus patients that allow many people with diabetes mellitus to attend in primary care units. The reason behind the negligence on self-care among the diabetes patient was low level of health literacy.

Health literacy includes speaking, listening, cultural knowledge and understanding of specific concepts that are necessary to interpret health information. ^6^ Hence, if people with diabetes mellitus have sufficient health literacy, they will be able to assist patients for self-care and blood sugar control to overcome the further complications. Therefore, the causal reasons of T2DM are confirmed by several studies involving with health-related factors of individuals such as healthy diet and exercise habits playing a key role in dealing glycemic control. ^7^^–^^9^

Low health literacy is common among individuals with poor health behaviors. ^7^^–^^9^ A study including diabetes related conditions and its controlling mechanism inducing health literacy has yet not been explored in Thailand. So, this study aimed to identify the health literacy among patients with T2DM who are treated in primary care units. As primary care unit taking care of the abovementioned patients, this study has played vital role to enhance quality care on controlling T2DM.

## Methods

### Ethics statement

Ethical approval for this study was granted by Nakhon Ratchasima Rajabhat University, under the testimonial number HE - 035 – 2561. Written informed consent to participate was provided by each of the study participants. Before collecting data, the researchers informed the participants about the study objectives and also took written informed consent for the publication of the research findings. Participants provided their signature on a consent form before the researchers collected data individually.

### Study design

This cross-sectional study aimed to examine health literacy among patients with T2DM, and identified factors associated with blood sugar control in the patients who were treated in primary care units in Nakhon Ratchasima province in Northeastern Thailand. The patient recruitment and data collection took place between July – November 2018. Adults ≥ 18 years of age diagnosed with type 2 diabetes mellitus for 1 year and more and living in Northeastern Thailand for 1 year and more were included in this study. However, adults <18 years of age and patients with type 1 diabetes, impaired glucose tolerance, metabolic syndrome, maturity onset diabetes of youth, and gestational diabetes as well as those who refused to give informed consent were excluded.

The present study was a cross-sectional study aimed to identify association of factors and blood sugar level control in T2DM patients who were treated in primary care units in Nakhon Ratchasima Province. Sample size requirement of the study was calculated using the standard sample size formula under multiple logistic regression ^10^ with the anticipated 80% of power of test and the 95% confidence interval arising from 2-side under the hypothesis testing. ^11^ After replacing the value in formula outcome (n=604.41). Thus the total number of samples taken for the study was 605.

### Study area

**Figure 1.**
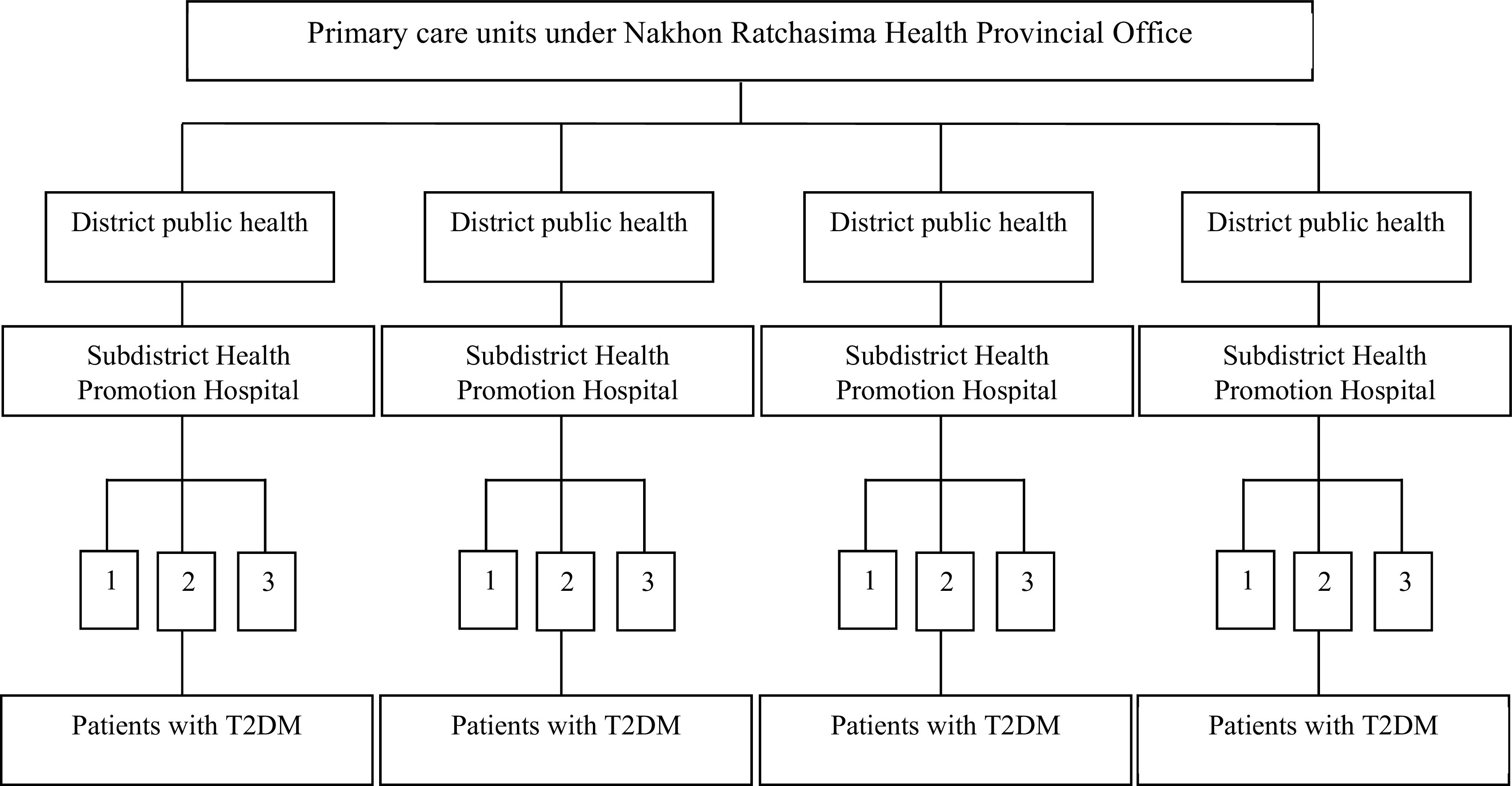
Procedure of multi-stage sampling among Type 2 Diabetes Mellitus (T2DM) patients.

The stratified and multi-stage sampling methods were applied to select the primary care units in Nakhon Ratchasima province. Of the total 32 districts in that province, four districts were selected by applying a simple random sampling method. After that, three sub-district health promotion hospitals were selected from each districts respectively. Finally, researchers proceeded to apply for permission from the 12 selected hospitals to collect the data among patients with T2DM who were treated in primary care units to complete the specified number of the samples shown in Figure 1.

### Instruments

In this research, data was collected by a structured questionnaire (available in *Extended data* ^35^) concerning health literacy and factors associated with blood sugar control among patients with T2DM. The questionnaire consisted of 4 parts; part 1 focused on personal characteristics such as sex, age, marital status, family history, duration of diabetes mellitus, cigarette smoking, alcohol drinking, obesity, education level, occupation, and income. Part 2 was an assessment form of health behavior. part 3 was an assessment form of health literacy among patients with type 2 diabetes mellitus, and was divided into 6 sectors following Nutbeaming D. ^12^ including 1) Access to health information and health services for diabetes mellitus 2) Knowledge and comprehension of diabetes mellitus 3) Communication of diabetes mellitus 4) Diagnosis of diabetes mellitus 5) Self-management of diabetes mellitus and 6) Source of getting information of diabetes mellitus. Lastly, part 4 comprised an assessment form of blood sugar control by evaluating blood sugar control among patients with T2DM in primary care units. Fasting blood sugar (FBS) and the sum of the blood sugar level of patients was recorded by a vocational nurse in the primary care units as part of routine procedure. The researchers then reviewed these patient charts using an assessment tool (the tool is provided at the end of the questionnaire in *Extended data* ^35^). Moreover, the researchers only collected data of blood sugar level among patients with T2DM who had been examined at primary care units three times. The mean of three times retrospective analysis of FBS level less than or equal to 154 mg/dl was considered as ‘controlled’ blood sugar level. Similarly, the retrospective analysis of three times FBS level more than or equal to 155 mg/dl was considered as ‘uncontrolled’ blood sugar level which has been based on the rigorous criteria for blood sugar control of Diabetes Association of Thailand. Generally, the purpose of blood sugar control is the accumulated amount of sugar in the blood or glycated hemoglobin (HbA1C) less than 7 (HbA1C < 7.0%) compared to the value of FBS not more than 154 mg/dL. ^13^

The research instrument was verified by the researchers who developed the questionnaire. After the formulation of the questionnaire three external experts validated the questionnaire by checking the consistence comprehensively. No changes were made to the questionnaire as a result of the verification and validation. The experts were specialized in the field of health literacy, health education and chronic disease control. The Item-Objective Congruence (IOC) was used to evaluate the item of the questionnaire. Every questionnaire has IOC up to 0.67.

The knowledge and comprehension and decision of diabetes mellitus questionnaire have KR-20 value equal to 0.76 and 0.74, respectively. The health behavior, accessing to health information and health service, communication, self-management, and media literacy provide information of diabetes mellitus questionnaire have Cronbach's alpha coefficient value equal to 0.73, 0.75, 0.74, 0.75 and 0.74, respectively.

### Data collection

Researchers collected data from the study participants who were T2DM patients in primary care units in Nakhon Ratchasima province through a questionnaire about health literacy and factors associated with blood sugar control among patients with T2DM. The questionnaire was administered in person at each of the primary care units. Additionally, data of various blood sugar levels of samples was collected via the questionnaire.

### Data analysis

This study aimed to collect quantitative data and conduct statistical analysis. Data were entered in excel and transferred to STATA (Version 13, Stata Corporation, College Station TXUSA) for analysis. Also, data analysis was separated into descriptive statistics to elucidate personal characteristic of samples involving sex, age, marital status, family history, duration of diabetes mellitus, consumer behavior, cigarette smoking, alcohol drinking, obesity, education level, occupation, and income including health literacy presented by frequency, percentage, mean, standard deviation, median, minimum, and maximum. In addition, inferential statistics used multiple logistic regression analysis to seek factors associated with blood sugar control among patients with T2DM by using bivariate analysis in case of categorical variables both polytomous and ordinal.

Furthermore, odds ratio (OR) along with 95% Confidence Interval and p-value were presented. In addition, variables with the p-value less than or equal to 0.25 or those variables which were mentioned significant in literature review were included for the multiple logistic regression. The backward elimination method was applied with the p-value <0.05 to obtain the result of multiple logistic regression. Finally, adjusted odds ratio (AOR) together with 95% CI of AOR and p-value were presented. ^14^

## Results

### Participant characteristics

The questionnaire data underlying the results are available in *Underlying data*. ^33^^,^^34^ The study found that patients with T2DM who attended primary care units in Nakhon Ratchasima province were mostly female (71.40%), of mean age 61.42 years (S.D.=12.04). Mostly, participants were aged between 60-79 years (50.74%). The maximum age was 90 years, and the minimum was 36 years. A total of 453 people (74.88%) were married. 58.02% of the respondents had attended primary school. In addition, 29.10% (176) respondents were unemployed or housewives. 333 (55.04%) of the respondents had income between 5,001-10,000 baht per month.

Similarly, 64.13% of the respondents were suffering from diabetes mellitus for 6-10 years. (46.28%) of the participants had normal diet behaviors. Simultaneously, 78.84% of the respondents were also practicing regular exercise. Furthermore, (69.26%) of the respondents were not smoking. Likewise,72.89% were not consuming alcohol and (65.62%) of the respondents had ideal weight.

### Health literacy among patients with type 2 diabetes mellitus

Health literacy analysis among patients with T2DM who attended primary care units in Nakhon Ratchasima province showed that almost all patients had health literacy at sufficient level (550 participants, 90.90%). A total of 55 participants had insufficient level of knowledge, as shown in Table 1.

**Table 1.** Display of health literacy levels among patients with type 2 diabetes treated in primary care units (n=605).

Health literacy level	Number (people)	Percentage (%)
Sufficient level (points value 43.00-70.00 points)	550	90.90
Insufficient level (points value 20.00-42.00 points)	55	9.10
Mean=50.71, S.D.=1.019 Max. : Min.=70: 26		

### Factors associated with blood sugar control

The bivariate analysis indicated that age, occupation, income per month, diet behavior, exercise behavior and health literacy were significantly (p-value <0.25) associated with blood sugar control among patients with T2DM who were treated in primary care units, as shown in Table 2.

**Table 2.** Frequency of blood sugar control and factors associated with blood sugar control among patients with type 2 diabetes mellitus treated in primary care units (Bivariate) (n=605).

Factors	Number (people)	Percentage of patients could control blood sugar levels	Odds ratio (OR)	95% CI of OR	p-value
Gender					0.352
Male	173	38.15			
Female	432	41.67	1.18	0.83-1.10	
Age					0.153
≥ 80 years old	46	26.09			
60-79 years old	307	41.69	2.03	1.01-4.06	
40-59 years old	210	42.86	2.13	1.04-4.33	
< 40 years old	42	35.71	1.57	0.63-3.92	
Marital status					0.282
Single	53	32.08			
Married	453	42.16	1.54	0.84-2.83	
Widowed/Separated	99	37.37	1.26	0.62-2.56	
Education level					0.342
Elementary school	351	38.75			
Junior high school	212	44.34	1.26	0.89-1.78	
Senior high school and above	42	35.71	0.87	0.45-1.71	
Occupational					0.214
Agriculture	171	48.54			
Private business	86	36.05	0.60	0.35-1.02	
Government officer	53	41.51	0.75	0.40-1.40	
Company staff	25	40.00	0.70	0.30-1.66	
General employment	94	38.30	0.65	0.39-1.09	
Housewife/not working	176	35.80	0.59	0.38-0.91	
Income per month					0.029
< 1,000 Baht	176	32.39			
1,001-5,000 Baht	333	45.65	1.75	1.20-2.57	
5,001-10,000 Baht	71	36.62	1.21	0.68-2.15	
10,001 Baht and above	25	40.00	1.39	0.59-3.29	
Hereditary of diabetes mellitus					0.476
No	388	39.43			
Yes	217	42.40	1.13	0.80-1.58	
Duration of diabetes mellitus					0.346
1-10 Years	438	38.81			
11-20 Years	130	43.85	1.23	0.82-1.83	
21 Years and above	37	48.65	1.49	0.76-2.92	
Diet behavior					<0.001
Fair	477	31.45			
Good	128	74.22	6.28	4.04-9.75	
Exercise behavior					<0.001
Never/sometimes	476	29.62			
Always	129	80.62	9.88	6.12-15.95	
Smoking status					0.230
Yes	164	36.59			
No	441	41.95	1.25	0.87-1.81	
Alcohol consumption					0.812
Yes	209	41.15			
No	396	40.15	0.96	0.68-1.35	
Body mass index (BMI)					0.790
Over	270	41.48			
Under	27	44.44	1.13	0.51-2.50	
Normal	308	39.29	0.91	0.65-1.27	
Health literacy					0.032
Insufficient level	55	27.27			
Sufficient level	550	41.82	1.92	1.03-3.55	

The multivariate analysis was performed to carryout multiple logistic regression where age, income per month, occupation and smoking status had shown significant association with blood sugar control and having good health literacy. The study participants who had sufficient health literacy could control blood sugar 2.27 times (AOR=2.27; 95%CI: 1.10-4.74; p-value<0.001) than that of those who had insufficient health literacy.

**Figure 2.**
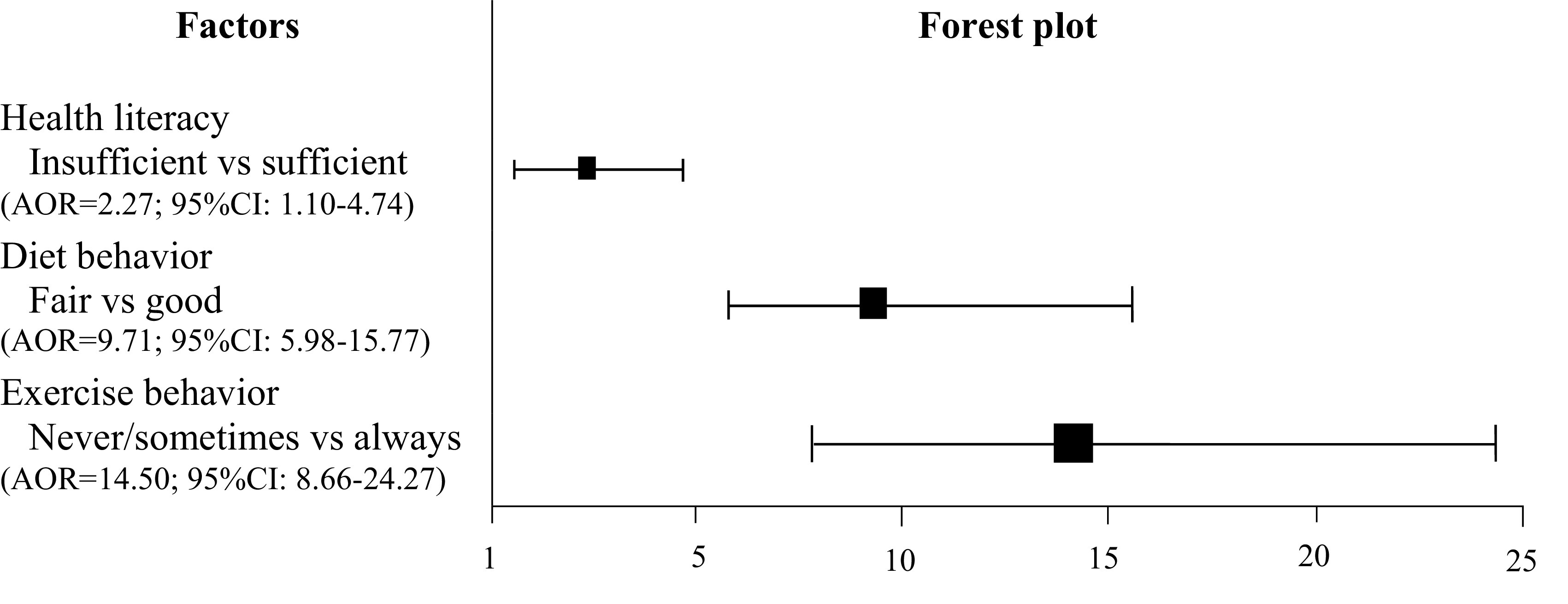
Adjusted Odds Ratios (AOR) and 95% Confidence Intervals (CI) for factors associated with blood sugar control among Type 2 Diabetes Mellitus (T2DM) patients treated in primary care units (Multivariate) (n=605).

Besides, analysis has shown exercise behavior could regularly control blood sugar 14.50 times (AOR=14.5; 95%CI: 8.66-24.27; p-value<0.001) when compared to people who never or sometimes exercised including diet behavior in good level could control blood sugar 9.71 times (AOR=9.71; 95%CI: 5.98-15.77; p-value<0.001) of diet behavior in fair levels, as shown in Figure 2.

## Discussion

In this present study, our purpose was to determine what factors of health literacy and related behaviors can be effective in controlling blood sugar level in patients with Type 2 Diabetes Mellitus (T2DM). Our result found almost half (41.82%) of the patients had sufficient health literacy among the T2DM patients and they were controlling their blood sugar level efficiently. The result showed patients with the sufficient health literacy had better outcome than those with the insufficient health literacy. The finding from multivariate analysis showed a significantly increased odds ratio of the association (AOR=2.27; 95%CI=1.10-4.74; p-value<0.05). This indicates that factor involves a disease complication when patients are unable to fully understand their ongoing disease. The patients needed to have a thorough understanding of T2DM as a key related factor that is essential to self-management for successfully controlling their glucose levels and ability to spend their life with T2DM properly. This could be recommended that health literacy is one of the key factors which can help to overcome T2DM patients in an effective manner.

A study towards chronic disease in people aged between middle age and older ^15^ revealed that people with a low health literacy may be impacted in their successful self-management this might be due to lack of knowledge and understanding on their decision-making, self-care, and treatment. Therefore, active participation of patients in the health information programs played important role for quality care. On the other hand, a recent study ^16^ denoted that diabetes patients still relied on support from their healthcare provider to control and treat their diabetes among patients who did not know enough about the target of blood glucose levels for diabetes management. These indicate that a sufficient knowledge of glycemic control is very important in controlling blood sugar levels.

However, in the context of whole primary health care service in Nakhon Ratchasima Province, T2DM information were provided for the patients through health education by health professionals when patients returned to follow-up visits. The information is combined both verbal and non-verbal methods of education information, for instance banners, pamphlets and inquiring to consultation with healthcare provider via LINE group. ^17^ This approach has also been established by few other studies, ^18^^–^^21^ which shows that the literacy is further increased because of the patient has an opportunity to interact with the respective diseases and get educated. ^22^

This study also showed that diet behavior among T2DM patients played a highly significant role in blood sugar levels control with increased odds ratio in the model of multivariate analysis (AOR=9.71; 95%CI=5.98-15.77; p-value<0.001). We assumed that the patients having a good diet behavior could have managed blood sugar levels than those having a fair diet behavior. Several previous studies ^23^^–^^25^ documented that people with T2DM can prevent diabetes complications when they are able to manage diets. It was observed by elevating HbA1c levels which were considered to be one of the leading risk factors for developing complications of blood vessels. The achievement then can be gained through dietary management. There is reported that food knowledge and understanding are key factors influencing diet behavior. The dietary choices and dietary habits of T2DM may be affected by adequate literacy and recommendations of the diet. A significant positive association was found between the knowledge of diabetic diet and the need for calories. It also concluded that knowledge of diabetic diet is essential and necessary for better dietary habits.

A similar finding found a significant odds ratio of very high magnitude in exercise behavior (AOR=14.50; 95%CI=8.66-24.27; p-value<0.001). The study showed the patients who had good exercise behavior had better blood sugar levels control than those with poor exercise behavior. This is supposed that exercise benefit is likely to show metabolic benefits for T2DM patients with stable and good blood sugar levels control. ^26^ This finding also confirmed by other studies ^27^^–^^29^ involving to glycemic control revealed a significant association with adherence of exercise beyond a healthy diet. There indicated that good diabetes control was significantly higher in T2DM patients who exercised regularly and a study ^30^ reflected a lack of glycemic control in only 21.0% of patients with HbA1c <7.0%. However, there is limitation to this part of exercise, it is not clear whether the finding was due to types of exercise habit. It seems reasonably clear that aerobic exercise is likely to be more effective than flexibility and other exercises. An explicit experimental study ^31^ on health benefits from intensive lifestyle interventions showed that aerobic exercise significantly improves glycemic control in adults with T2DM. This is especially true when performing at least 150 minutes per week. While free weight or weight machines improved HbA1c by 0.6%. Further empirical research is needed to resolve this uncertainty.

Nonetheless, the recommendation is that T2DM patients with poor exercise should add an unstructured physical activity every day to gain additional health benefits. Usual exercise thus helps to lower blood sugar levels for managing diabetes as muscles use sugar (glucose) for energy. However, a noticeable study ^32^ denoted that even light activities of daily life such as housework, gardening, or long walks can improve blood sugar levels and using energy for everyday activities can create a huge daily calorie burn.

## Data availability

### Underlying data

Patient records from the hospital were reviewed to enhance the quality of collected data. The chart assessment tool (Part 4 of the questionnaire) was developed to record (retrospectively) if the mean of three analyses of fasting blood sugar level (FBS) for each patient was less than or equal to 154 mg/dl (considered ‘controlled’ blood sugar level). Due to ethical and data protection concerns, we are unable to provide readers with access to the individual patient records from the hospital, as it will affect the anonymity of the individual. However, the data that was collected (i.e. whether the FBS level was considered ‘controlled’ or ‘uncontrolled’) has been uploaded along with the anonymized questionnaire data.

Zenodo: Health literacy and behaviors influencing blood sugar level control among type 2 diabetes patients in primary care units, Thailand: A Cross-Sectional Study.

https://doi.org/10.5281/zenodo.6325536. ^33^

This project contains the following underlying data:

- Final_Health literacy among type 2 diabetes patients in primary care units, Thailand.xlsx (questionnaire data).

Zenodo: Health literacy and behaviors influencing blood sugar level control among type 2 diabetes patients in primary care units, Thailand: A Cross-Sectional Study. https://doi.org/10.5281/zenodo.5914387. ^34^

This project contains the following underlying data:

- Data key or dictionary for Questionnaire_HL_DM.docx.

### Extended data

Zenodo: Health literacy and behaviors influencing blood sugar level control among type 2 diabetes patients in primary care units, Thailand: A Cross-Sectional Study.

https://doi.org/10.5281/zenodo.5864806. ^35^

This project contains the following extended data:

- Questionnaire_HL_DM_Final.docx (questionnaire tool).

Data are available under the terms of the Creative Commons Attribution 4.0 International license (CC-BY 4.0).
