# Supplementary material for: Health literacy and behaviors influencing blood sugar level control among type 2 diabetes patients in primary care units, Thailand: A cross-sectional study
Source: F1000Res. 2022 Mar 18;11:332. [Version 1] doi: 10.12688/f1000research.74225.1 (PMC10730987; doi:10.12688/f1000research.74225.1)
Supplement: Peer review history

# Reviewer report: Reviewer response for version 1

**DOI:** 10.5256/f1000research.77956.r199342

**Published:** 2023-12-19

**Authors:** Harry H X Wang

**Affiliations:** 1 School of Public Health, Sun Yat-Sen University, Guangzhou, Guangdong, China

Singsalasang and colleagues conducted a cross-sectional study to explore the association of health literacy and behaviours with blood glucose control in primary care patients with diabetes in Thailand. The study provides evidence suggesting the need for health practitioners to enhance patients' health literacy on self-care. 

 Reference #1: This is a local report in Thailand, which may not sufficiently support the background description on the contribution of type 2 diabetes (T2D) to mortality at a global level. The authors also provided statistics from the International Diabetes Federation (IDF), yet the IDF report was not cited appropriately in the Reference section. 

 It appears that the study excluded patients with metabolic syndrome. As diabetic patients may often have other comorbidities than diabetes alone, the exclusion of diabetic patients with concurrent components of metabolic syndrome may lead to selection bias. This could be acknowledged as a study limitation.

 Some additional reference might be helpful for international readers to better understand the calculation process of Item-Objective Congruence (IOC) and KR-20 value.

 The authors may wish to briefly describe how to ensure data accuracy and completeness during data entry. 

 In Table 1, it is not clear how the cut-off point of the health literacy level was derived.

 Figure 2 shows that health literacy level and diet and exercise behaviours serve as significant factors associated with blood glucose control. However, there is a lack of essential description on how diet and exercise behaviours were measured in the Methods section in the manuscript.

Is the work clearly and accurately presented and does it cite the current literature?

Partly

If applicable, is the statistical analysis and its interpretation appropriate?

Yes

Are all the source data underlying the results available to ensure full reproducibility?

Yes

Is the study design appropriate and is the work technically sound?

Yes

Are the conclusions drawn adequately supported by the results?

Yes

Are sufficient details of methods and analysis provided to allow replication by others?

Partly

Reviewer Expertise:

Primary Health Care

I confirm that I have read this submission and believe that I have an appropriate level of expertise to confirm that it is of an acceptable scientific standard.

# Reviewer report: Reviewer response for version 1

**DOI:** 10.5256/f1000research.77956.r225060

**Published:** 2023-12-15

**Authors:** Cecilia Jimeno

**Affiliations:** 1 Division of Endocrinology, Diabetes and Metabolism, Department of Medicine, Philippine General Hospital & Department of Pharmacology and Toxicology, College of Medicine, University of the Philippines, Manila, Philippines

**Comments**

  

1. The title can be improved to, “A Cross Sectional Study on Healthy Literacy and Behaviors influencing Glycemic control among patients with Type 2 diabetes in Primary care Units in Thailand”
2. Abstract- a little long at 288 words
3. Key words- may be better to use “glycemic control” instead of “blood sugar level control”
4. Introduction
  1. The introduction must include a brief background of how health literacy is measured- what aspects are measured and how- and if a particular tool was adapted for purposes of this study
5. Materials and Methods
  1. How was Part 3- which is the Questionnaire on Health Literacy developed? Was it adapted from an existing questionnaire? Was it validated and pilot tested before using in this study? Was it made available in the local language or dialect? How were the number of questions and the specific questions to developed? How is it scored?  In short, there needs to be a brief discussion on questionnaire development. While there is some write-up on the validation part, there are still some uncertainties. For example, what was considered to be an acceptable IOC?
  2. What is the KR value of the questionnaire? What does KR mean, what does it measure and what is the ideal value?
  3. How was the minimum pass level of 43-70 points for the Health Literacy questionnaire decided on? This corresponds to around 61-100% as being considered passing
  4. The cut-off value of 154 mg/dL is a little high and does not really correspond to an HbA1c value of 7.0%. The 150 mg/dL cut-off value there is the average glucose, which is the summation of both fasting and post-prandial values, not the average fasting glucose. To reach an HbA1c of 7.0%, one needs to aim for a lower FPG than 154.
6. Results
  1. The BMI should be stated as 4 categories: Underweight, Normal, Overweight (not just “over”) and Obese
7. Limitations: The cut-off for good blood sugar is really much higher than is acceptable and thus, becomes a major limitation in interpreting the results of this study. 
8. Improve grammar and writing style. For example, in the introduction, the word “raised” is used in 2 consecutive sentences [It has been estimated that the prevalence of diabetes mellitus will be unraised and constant at 6.90% by the year 2025. The mortality statistics also emphasized that the T2DM will be raised in future despite the trend of subsiding the non-communicable diseases.] However, the correct word I think is not “unraised” or “raised” but increase e.g.. the prevalence of diabetes mellitus will not increase.

Is the work clearly and accurately presented and does it cite the current literature?

Yes

If applicable, is the statistical analysis and its interpretation appropriate?

Yes

Are all the source data underlying the results available to ensure full reproducibility?

Yes

Is the study design appropriate and is the work technically sound?

Yes

Are the conclusions drawn adequately supported by the results?

Yes

Are sufficient details of methods and analysis provided to allow replication by others?

Partly

Reviewer Expertise:

Endocrinology, diabetes, pharmacology, thyroid epidemiology

I confirm that I have read this submission and believe that I have an appropriate level of expertise to confirm that it is of an acceptable scientific standard, however I have significant reservations, as outlined above.

# Reviewer report: Reviewer response for version 1

**DOI:** 10.5256/f1000research.77956.r128026

**Published:** 2022-03-22

**Authors:** Kunwadee Rojpaisarnkit

**Affiliations:** 1 Department of Public Health, Faculty of Science and Technology, Rajabhat Rajanagarindra University, Chacheongsao, Thailand

1. It should be clearly stated that the health literacy in this study is the health literacy of glycemic control in diabetic patients.
2. Study design: should review the writing about the exclusion criteria of sample. This is because those under the age of 18 and with type 1 diabetes should not be in the inclusion criteria at the outset. Therefore, it does not need to be specified as an exclusion criteria.
3. Figure 1 should increase the number of samples clearly. And in the description of the diagram, it should be clearly stated that “What are reasons for choosing 4 areas at the district level, and choosing 3 sub-district health promoting hospitals in each district to collect data?”.
4. Instrument: The number of questions in each part of the questionnaire should be clearly stated.
5. Research methodology: In the analysis of the data, it should be clearly stated how the backward method was analyzed for multiple logistic regression (backward stepwise: likelihood ratio or backward stepwise: wald or backward stepwise: condition).
6. Data collection: It should be clearly stated how the glycemic data was collected using the investigator questionnaire. Ask the patient or not? (Does the reviewer understand that the data is collected from the patient treatment record form or not?).
7. The sixth line of the Results section relates to participant characteristics. There is erroneous information indicating that 55.40% of the sample has income between 5,001-10,000. From the data in Table 1, it is found that 55.40% of sample has income between 1,001-5,000 baht.
8. The results showed that the respondents had health literacy at a sufficient level of 90.90%. Researchers should discuss why and how it differs from the overall research findings on the health literacy level of the people in Thailand, and why is that?
9. The results showed that the personal characteristics of the sample, 55.40% had income between 1,001 -5,000 baht, which is lower than the average minimum income of Thai people, and 58.02% had an education level at the primary school level, indicating that most of the samples were in the low socioeconomic group. Why did this group of people have a sufficient level of health literacy? Researchers should clearly discuss these findings.
10. In addition, the results of the study revealed that the health literacy at the sufficient level of the samples still does not affect some health behaviors. For example, from Table 1, most of the samples did not exercise and almost half still have alcohol consumption behavior, researchers may increase the discussion on these issues.
11. Table 2 shows that the percentage of the sample who can control their sugar levels, there are some variables that can be noted as observations from the study. For example, people with normal, above-normal, and lower-than-normal BMIs have a similar percentage of glycemic control or people who drink and do not drink alcohol have a similar percentage of sugar control, etc.
12. According to the results of the analysis of the relationship between individual attribute variables and blood sugar level, it is stated that researchers using the multiple logistic regression statistics. In this part of the analysis, should the binary logistic regression analysis be used or not? (because there is only one dependent variable), please describe your reason.

Is the work clearly and accurately presented and does it cite the current literature?

Partly

If applicable, is the statistical analysis and its interpretation appropriate?

Partly

Are all the source data underlying the results available to ensure full reproducibility?

Yes

Is the study design appropriate and is the work technically sound?

Yes

Are the conclusions drawn adequately supported by the results?

Partly

Are sufficient details of methods and analysis provided to allow replication by others?

Partly

Reviewer Expertise:

Public health, Health Science

I confirm that I have read this submission and believe that I have an appropriate level of expertise to confirm that it is of an acceptable scientific standard, however I have significant reservations, as outlined above.
